# Attentional processing in individual anxiety: Electrocortical correlates of threat- and safety-related declarative memory formation

**DOI:** 10.3758/s13415-026-01450-0

**Published:** 2026-05-12

**Authors:** Yannik Stegmann, Valentina Glück, Marta Andreatta, Julian Wiemer

**Affiliations:** 1 Department of Psychology, University of Würzburg, Marcusstraße 9-11, 97070 Würzburg, Germany; 2 Department of General Psychiatry and Psychotherapy, University Hospital Tübingen, Tübingen, Germany; 3 Tübingen Center for Mental Health, University Hospital Tübingen, Tübingen, Germany

**Keywords:** Memory, Conditioning, Event-related potentials, Anxiety, Anxiety sensitivity index

## Abstract

The ability to accurately detect and remember threatening stimuli is essential for adaptive behavior in potentially dangerous environments. While threat-related defensive processes are increasingly well understood, recent research has begun to emphasize complementary safety-related attention and learning mechanisms, processes especially relevant to the development and maintenance of pathological anxiety. Prior fear conditioning studies have identified a key role for late positive event-related potentials, particularly the P300 and late positive potential (LPP) in the formation of declarative memories related to threat and safety. However, the influence of individual differences in anxiety on these mechanisms remains insufficiently explored. In the present study, we examined how anxiety sensitivity, a transdiagnostic risk factor for anxiety-related pathologies, modulates attention and memory processes during declarative threat and safety learning. Using a subsequent memory paradigm, we recorded event-related potentials as participants learned associations between neutral faces and either an aversive outcome or safety. Stronger P300 and LPP amplitudes were found for remembered compared with forgotten items. Crucially, elevated anxiety sensitivity was associated with increased P300 and LPP amplitudes at the onset of both threat and safety cues, indicating heightened attentional allocation to both types of stimuli. Despite these amplified neural responses during memory encoding, higher anxiety sensitivity did not correspond to enhanced memory performance. These results suggest that, while individuals at heightened risk for anxiety-related psychopathology exhibit generalized hypervigilance, this increased attentional engagement does not necessarily translate into enhanced memory formation during intentional learning.

**Supplementary Information:**

The online version contains supplementary material available at https://doi.org/10.3758/s13415-026-01450-0.

## Introduction

Associative learning is a fundamental mechanism that enables organisms to navigate dangerous environments effectively. The ability to learn and predict potential dangers is crucial for survival, because it allows organisms to avoid harmful situations (Fanselow, 2018). Equally important, however, is the capacity to recognize and predict periods of safety, because this facilitates adaptive behaviors, such as resting, foraging, and social interactions. An imbalance between these processes is discussed as a key factor for the development of anxiety disorders (Mineka & Oehlberg, 2008). A bias towards threat can lead to excessive fear responses that are accompanied by hypervigilance for threat-related stimuli and persistent avoidance behavior (Beckers et al., 2023). Thus, individuals must ensure that information about both threats and safety is correctly retained over time and can be effectively retrieved when encountering similar situations.

In the laboratory, these processes are usually studied with Pavlovian fear conditioning paradigms, in which an initially neutral stimulus (CS +) is repeatedly paired with an inherently aversive stimulus (US), such as a mildly painful electric stimulation, while another neutral stimulus (CS −) is never paired with the US (Lonsdorf et al., 2017; Pavlov, 1927). Over time, individuals learn to respond defensively to the CS + as it predicts danger, but not to the CS − as this stimulus becomes associated with threat absence, which is defined as safety (Seligman & Binik, 1977). Eventually, the presentation of the CS + alone is able to elicit a defense reaction characterized by an adaptive response pattern involving multiple systems. On a physiological level, the presentation of the CS + usually triggers heightened skin conductance (Ojala & Bach, 2020), increased pupil dilation (Korn et al., 2017), and a reduction in heart rate (fear bradycardia; Battaglia et al., 2022), all mediated by the autonomous nervous system. In humans, these physiological changes are often accompanied by feelings of distress or fear (Lang et al., 2000). Additionally, the attentional system prioritizes processing of the threat-related stimulus, showing heightened vigilance toward the CS + (Miskovic & Keil, 2012; Wieser & Keil, 2020).

Alongside identifying the fundamental mechanisms of defense responses, Pavlovian fear conditioning paradigms have been proposed as a model for understanding of clinical anxiety (Beckers et al., 2023; Craske et al., 2009), as the processes involved in the acquisition of conditioned fear may closely parallel the mechanisms contributing to the development of pathological fear and anxiety. In this perspective, it is assumed that individuals that are at risk of developing clinical anxiety also tend to show stronger defensive responses in a fear conditioning paradigm, such as enhanced physiological reactions, or are faster in acquiring conditioned fear responses. Meta-analytical evidence, however, challenges the idea that anxiety patients exhibit stronger fear responses to the CS + compared with healthy controls (Kausche et al., 2025). Instead, these studies highlight that a key distinction between clinical anxiety and healthy controls lies in heightened responses to the CS −, which has been interpreted as a deficit in safety learning. Despite being a focal point of recent fear conditioning research, the neural mechanisms underlying deficient safety learning in clinical anxiety remain elusive. Earlier theories suggested that individuals with pathological anxiety exhibit heightened attentional sensitivity to potentially threatening cues in general and tend to adopt a “better-safe-than-sorry” strategy, resulting in enhanced fear responses to inherently neutral stimuli or conditioned safety signals (Wiemer & Pauli, 2016a;2016b). At the same time, individuals at risk for anxiety disorders may be more prone to overgeneralizing conditioned fear, as indicated by heightened fear responses to ambiguous cues that perceptually or conceptually resembles the original CS + despite never having been directly paired with the US (Cooper et al., 2022; Fraunfelter et al., 2022). Prevailing learning accounts view safety learning as an active construction of an association between stimulus and safety, which is more complex than a mere weakening of an association between stimulus and threat (Craske et al., 2014). Aberrances in safety learning in anxiety disorders could, thus, reflect an impaired ability to actively inhibit fear in the presence of safety cues. Crucially, these processes (i.e., fear acquisition, generalization, and safety learning) are thought to be highly interrelated, potentially contributing to the development of anxiety disorders through shared mechanisms (Stegmann et al., 2019b).

As outlined above, fear conditioning studies typically quantify conditioned responses and associative memory through psychophysiological or neural indicators of the defense response, often overlooking the role of more complex cognitive processes such as declarative memory. Declarative memory refers to the explicit, conscious recall of facts and events, distinguishing it from the more automatic, implicit associations typically studied in conditioning paradigms. This distinction is particularly important, as psychotherapy primarily targets the explicit components of fear and anxiety rather than the implicit processes emphasized in traditional conditioning research (Hofmann, 2008). To fill this gap, we recently adapted a subsequent memory paradigm to fear conditioning and explored the electrocortical (Leimeister et al., 2023; Wiemer et al., 2021) and neural (Wiemer et al., 2024) correlates of declarative memory formation. To this end, participants were shown multiple faces, half of which (CS +) were associated with the US and half of which were not (CS −), while brain activity was recorded via EEG or fMRI. Declarative memory was assessed in a subsequent recognition task. By comparing brain responses to remembered associations with those to forgotten associations, it is possible to identify neural activity linked to successful memory encoding during fear and safety learning (Paller & Wagner, 2002). Crucially, we deliberately focused on memory for CS outcome associations rather than on old–new recognition memory. Our goal was to examine how attentional and evaluative processes during threat and safety learning contribute to the formation of declarative memories linking cues to aversive outcomes or their omission. While old–new recognition paradigms have provided important insights into threat- and safety-related memory processes (Weymar et al., 2014; 2019; Ventura-Bort et al., 2016; Schellhaas et al., 2022; 2020), the present approach was chosen to specifically target associative learning mechanisms. Our previous findings robustly revealed an enhanced late positive potential (LPP) during CS + and CS − processing. Beyond neural responses elicited by the CSs themselves, responses to CS outcomes are also likely to contribute to associative fear and safety memory formation. In particular, encoding the absence of threat may be a critical mechanism underlying successful safety learning. As prediction error signals are widely considered central to associative learning in both reward and aversive domains (Li & McNally, 2014), we additionally examined electrocortical activity to the outcome at the offsets of the CSs. These analyses revealed larger P300 amplitudes in response to the US (at CS + offset) and in response to the omission of the US (at CS − offset) for remembered compared to forgotten associations. As the P300 and LPP are established indicators of attention allocation (Hajcak & Foti, 2020; Hajcak et al., 2010), these results support the notion that attentional allocation to the outcomes plays a crucial role in declarative threat and safety memory formation. This idea is further substantiated by findings from our fMRI study (Wiemer et al., 2024), which demonstrated generally higher activity in hippocampal regions for associations that were later remembered vs. forgotten. In contrast, activity in the ventromedial prefrontal cortex predicted subsequent memory during safe associations (between CS − and US omission responses), whereas activity in the left dorsolateral prefrontal cortex was predictive for outcomes in general (US presentations and omissions). Combined, these findings from both the EEG and the fMRI studies highlight the interplay between prefrontal and sensory areas as a central mechanism in the formation of declarative safety memory.

These findings on neural underpinnings of safety learning in healthy populations may help to elucidate aberrances in the attentional processing of safety signals in pathological anxiety as well. However, how high levels of individual anxiety influence attention and memory mechanisms related to declarative safety learning needs yet to be explored. One approach to address this gap is to examine both individuals diagnosed with clinical disorders and those at risk of these disorders, specifically individuals who score high on psychometric assessments of psychopathology. In this context, the construct of anxiety sensitivity, defined as the belief that experience of anxiety causes illness, embarrassment, or additional anxiety (Reiss et al., 1986), is particularly useful, because it is considered a transdiagnostic factor across various forms of psychopathology (Allan et al., 2014a; Boswell et al., 2013; Deacon & Abramowitz, 2006; McNally, 2002) and can be measured dimensionally, making it well-suited for studying individual differences in anxiety.

Consequently, the purpose of the current study was to investigate the electrocortical correlates of memory formation among a sample varying in individual anxiety using the previously established aversive learning and memory paradigm. We hypothesized enhanced P300, LPP, and pupillary responses to CS onsets (both CS + and CS −), as well as enhanced P300 and LPP amplitudes to the CS − offset during learning, for subsequently remembered vs. forgotten stimuli. Furthermore, we expected that the differences in these three parameters between remembered vs. forgotten CS − will be reduced in participants with high levels of individual anxiety.

## Method

### Transparency and openness

All data and code behind the analyses have been made publicly available at the Open Science Framework and can be accessed at https://osf.io/83zbx/. This study was not preregistered.

### Participants

**Fig. 1:**
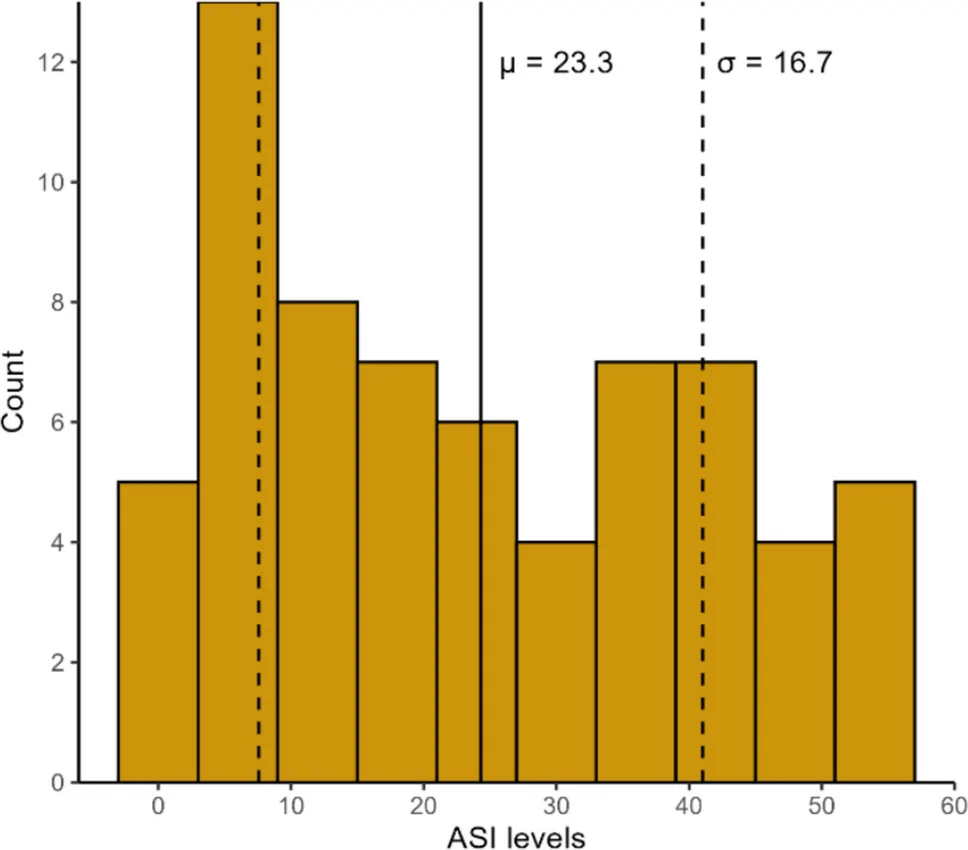
Distribution of ASI levels of the final sample (n = 66). *Note.* Mean and standard deviation scores are depicted as solid and dashed vertical lines, respectively

Participants underwent a two-step screening process with an online recruitment program from the University of Würzburg and local advertisements. As the first screening step, individuals filled in an online questionnaire with questions on self-reported panic attacks in the previous 2 weeks and the German version of the Anxiety Sensitivity Index (ASI-3; Kemper et al., 2009; Taylor et al., 2007). We specifically recruited participants with low and high levels of anxiety to cover the whole range of the anxiety spectrum. To this end, individuals with ASI sum scores below 18 or above 22 (cutoffs based on Allan et al., 2014b) and individuals who had indicated that they had experienced panic attacks in the 2 weeks prior to questionnaire participation were invited to a telephone interview. In this second screening step, we performed the semistructured interview Mini-DIPS (Margraf et al., 2017) to confirm the presence or absence of anxiety-related pathologies (i.e., diagnosis of current panic disorder, generalized anxiety disorder, social anxiety disorder, and/or agoraphobia as diagnosed in the interview) and the absence of further exclusion criteria (history of drug or alcohol misuse or dependency, neurological disorders, major depression disorder in the previous 6 months, use of antipsychotic medication, history of bipolar or psychotic disorders). In the end, we included also individuals with ASI-3 sum scores below 18 or above 22, if they did not fulfill any of the exclusion criteria. The screening procedure led to 87 participants taking part in the experiment. Of those, three participants did not complete the task, and 18 participants did not generate the sufficient number of 15 remembered/forgotten trials per condition (after excluding trials with EEG artifacts, see below). The final sample involved 66 participants (age: *M* = 25.4, SD = 6.5; 51 female). Of those, 16 participants reported a history of anxiety-related pathologies according to the Mini-DIPS interview (6 had social anxiety disorder, 9 generalized anxiety disorder, 4 panic disorder; 4 participants had more than one comorbid pathology; mean ASI = 38.6, *SD* = 16.5). The distribution of ASI levels is illustrated in Fig. 1. Due to the relatively complex screening procedure, the sample size was determined based on practical considerations (e.g., participant availability, time constraints) rather than a formal power analysis. The participants were compensated with course credit or 30 Euros. All participants had normal or corrected-to-normal vision. This study was approved by the ethics committee of the psychological department of the University of Würzburg (GZEK 2017–07). We report how we determined our sample size, all data exclusions (if any), all manipulations, and all measures in the study.

### Stimuli

#### Conditioned Stimuli (CS)

Visual stimuli were presented on a 19-inch monitor with a refresh rate of 60 Hz. We used 80 different black and white pictures of male and female neutral facial expressions as conditioned stimuli. The pictures were obtained from the Chicago Face Database (Ma et al., 2015). The black-and-white face images were displayed for 3 s each on a grey background (RGB = 144), designed to match the luminance of the faces. The pictures were separated into two subsets of 40 pictures each, one serving as CS + and one as CS −, within one session. The assignment to CS + and CS − was counterbalanced between participants. In addition, the two subsets were matched for the sex of the models, arousal, attractiveness, and luminance (all *p*s > 0.22).

#### Unconditioned Stimulus (US)

The aversive US was an aversive electrical stimulus applied to the inner side of the left calf via a current stimulator (Digitimer DS7A; Digitimer Ltd, Welwyn GardenCity, UK) and two steel surface electrodes (9-mm diameter; GVB-geliMED, Bad Segeberg, Germany). The electrical stimulation was set to 400 V for 40 ms (10 pulses of 2 ms stimulation with 2 ms breaks). Current intensity was individually calibrated to each participant’s pain threshold using a standardized procedure involving two ascending and descending series of electrical stimulations. The target was a subjective intensity rating of 4 (“just noticeably painful”) on a scale from 0 ("no noticeable sensation") to 10 ("extremely painful") was targeted. This individual US intensity was increased by 20% to obtain the final individual US intensity. Mean current intensity was *M* = 1.32 (*SD* = 1.31).

### Procedure

**Fig. 2:**
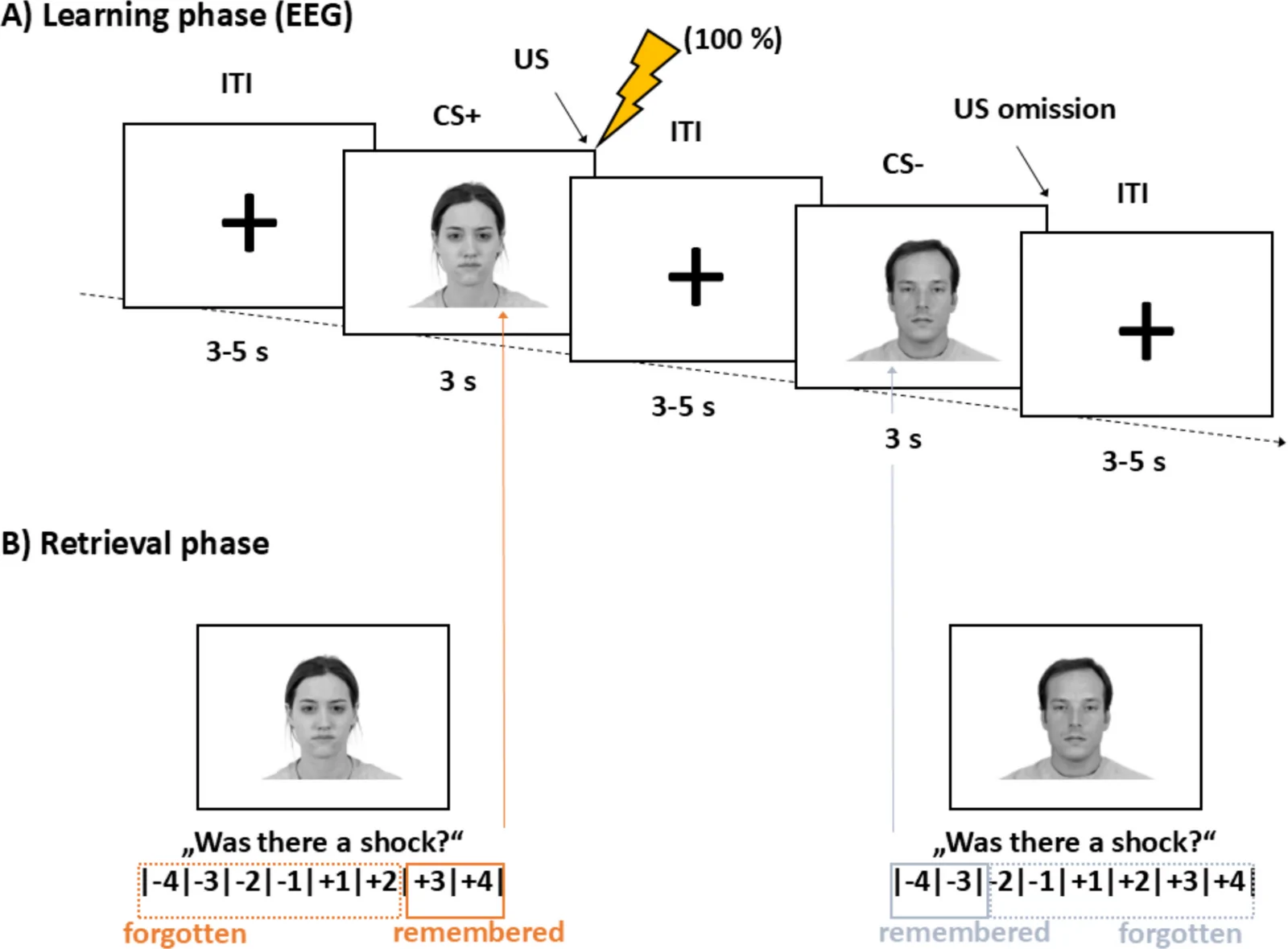
Design and trial structure of the main task. *Note.* **A**) EEG was recorded during the learning phase, in which every CS + was followed by an aversive electrical unconditioned stimulus (US). **B**) Conditioned stimuli (CS + and CS −) were categorized as remembered or forgotten, depending on memory ratings in the retrieval phase

First, participants provided informed consent and completed a sociodemographic questionnaire. They were then seated in a sound-attenuated, dimly lit testing room. EEG, and electrical stimulation electrodes were subsequently applied. The electrical stimulation was adjusted to each participant’s individual pain threshold and the eye tracker was individually calibrated for optimal capturing of the pupil dilation before beginning the experiment. The main experimental part consisted of two phases: a learning phase, and a retrieval task, following the design by Leimeister et al. (2023). The learning phase started with participants instructed to memorize the association between faces (CS) and electrical stimulation (US). A total of 80 faces were presented once in each of three consecutive blocks, with short self-paced breaks in between blocks. This resulted in every CS + and CS − being presented three times, for a total of 240 trials. Each face was displayed for 3 s. USs were delivered at the offset of the CS + face images (reinforcement rate was 100%, resulting in a total of 120 electrical stimulations). During the inter-trial-interval, a black fixation cross was displayed in the middle of the screen for a random duration of 3–5 s (Fig. 2).

After the learning phase, participants completed a 15-min visuo-spatial cognition task, which involved solving three-dimensional puzzles. This task was designed to prevent them from further engaging with the subsequential memory task.

Following this, participants underwent the retrieval task during which they viewed each CS again and were asked to indicate whether the given picture had been associated with a US. The confidence in their judgment was rated on an eight-point scale ranging from − 4 (very certain there was no US) to + 4 (very certain there was a US), excluding zero. Conditioned stimuli (CS + and CS −) were categorized as remembered or forgotten, depending on memory ratings in the retrieval phase: High confidence hits (+ 4 and + 3 for CS +; − 4 and − 3 for CS −) were classified as correctly remembered, while all other ratings were classified as forgotten (Leimeister et al., 2023; Wiemer et al., 2021, 2024). Additionally, participants provided valence and arousal ratings for each picture, using scales ranging from 0 (very unpleasant, very calm) to 100 (very pleasant, very intense).

### EEG recording and data preprocessing

EEG recordings were performed using a 32-channel system (ActiCap; Brain Products, Munich, Germany) with active Ag/AgCl electrodes placed according to the 10–20 system and a Brainamp amplifier (Brain Products, Munich, Germany). Data were recorded during the learning phase with reference to mastoid electrodes and filtered online between 0.01 and 250 Hz, with a sampling rate of 1,000 Hz. Electrode impedances were kept below 5 kΩ. Furthermore, an electrooculogram was recorded from two horizontal and vertical eye electrodes. Brain Vision Recorder Version 1.05 and ActiCap Control Software (Brain Products, Munich, Germany) were used for data collection.

EEG was analyzed offline using EEGlab v2022.1 (Delorme & Makeig, 2004). The data were first re-referenced to the common average and low-pass filtered using a 40 Hz Butterworth filter (transition band width = 10 Hz, cutoff-frequency = 45 Hz). Bad channels were excluded and interpolated (spherical spline method) automatically using the z-value criterion of z = 3.29 (Tabachnick & Fidell, 2007) for the probability and kurtosis on channel level. To correct for various artifacts, an ICA procedure and the EEGlab plugin IClabel (Pion-Tonachini et al., 2019) were used. Components with a probability ≥ 0.5 of being classified as either an eye artifact, muscle artifact, heart artifact, line noise, or channel noise were rejected. The data were then segmented into epochs between − 200 ms pre-stimulus and 1,000 ms post-stimulus and baseline-adjusted for a time window of − 200 to 0 ms. Separate segments were created for CS onsets and CS − offsets (i.e., US omission). Artifact rejection was based on an amplitude threshold (− 150 to + 150 µV) and a joint probability threshold (*SD* = 5). As mentioned above, participants were excluded if, after artifact rejection, fewer than 15 trials per condition (CS +/CS- x remembered/forgotten) were available for analysis, resulting in an exclusion of 14 participants. The remaining participants displayed an average (± SD) rejection rate of 7.5 ± 17%. Epochs were averaged for each of the following conditions: CS + remembered (retained trials: 50.2 ± 25.1; rejection rate: 5.8 ± 16%), CS + forgotten (retained trials: 60.7 ± 25.9; rejection rate: 5.6 ± 12%), CS − remembered (retained trials: 41.0 ± 22.0; rejection rate: 4.9 ± 13%), CS − forgotten (retained trials: 69.9 ± 23.3; rejection rate: 6.2 ± 13%), CS − offset remembered (retained trials: 38.2 ± 22.3; rejection rate: 8.8 ± 18%), and CS − offset forgotten (retained trials: 65.0 ± 25.8; rejection rate: 13.7 ± 26%). 

To stay consistent with our previous findings, both the P300 and the LPP were scored at centro-parietal electrodes (Schupp et al., 2000). Specifically, we extracted P300 and LPP responses to the cue onset over Pz, whereas cue offset responses were scored over Cz, as different topographies were obtained for cue onset and offset in the current and previous iterations of the task. For both regions, the amplitude of the P300 was calculated as the mean amplitude between 200 and 400 ms following the CS onset or offset, while the amplitude of the LPP was calculated as the mean amplitude between 400 and 1,000 ms following the CS onset or offset, similar to previous work (Leimeister et al., 2023; Wiemer et al., 2021).

### Pupil and SCR recording and preprocessing

Pupil diameter was recorded during the learning phase with a Tobii Pro Nano eye-tracker (Tobii Technology) at a sampling rate of 60 Hz. First, we performed a linear interpolation on segments containing blinks and missing data. Following this, we applied a 2 Hz low-pass filter and conducted a baseline correction by subtracting the mean of the − 500 to 0 ms time window, relative to the cue onset, from each ensuing data point. For the statistical analysis, we calculated the average pupil diameter between 0.5 and 3 s for every condition (Reutter & Gamer, 2023).

### Statistical analysis

Mean differences in ERP amplitudes, pupil diameter changes, as well as valence, arousal, and memory ratings were analyzed with linear mixed models with the within-subject factors CS-type (2 levels: CS + vs. CS −) and Memory (2 levels: forgotten vs. remembered) and mean-centered ASI scores as between-subjects covariate. All three main effects and their interactions were entered as fixed effects. Participants were entered as random intercepts to the models. LPP and P300 amplitudes to the US omission were analyzed similarly but without the factor CS-type. Significance was evaluated using the Kenward-Rogers approximation for degrees of freedom (Kenward & Roger, 1997; Luke, 2017). The alpha level was set at *p* < 0.05 (two-tailed). Linear mixed models were conducted in the R software environment (version 4.4.2.; R Development Core Team, 2021), using the package ‘lme4’ (version 1.1–36; Bates et al., 2015). Standardized effect sizes and 95% confidence intervals (95% CI) for the discrete factors of the linear mixed models were calculated as partial-*R*^*2*^, using the package ‘r2glmm’ (version 0.1.2; Jaeger et al., 2017). For visualization purposes only, anxiety sensitivity was median-split to illustrate group-level patterns in figures. Importantly, all statistical analyses were conducted using continuous anxiety sensitivity scores. No inferential conclusions were based on the median-split grouping, and all reported effects reflect models treating anxiety sensitivity as a continuous variable. In addition to this dichotomized memory analyses, we also conducted exploratory analyses treating US-memory ratings as a continuous variable. Linear mixed-effects models were specified analogously to the primary analyses but included continuous memory ratings instead of categorical remembered-versus-forgotten contrasts. Due to substantial variability in the number of analyzable trials across conditions and participants, as well as the presence of empty cells in some memory × CS-type combinations, these analyses were considered exploratory and are reported in detail in the supplemental material.

## Results

### Ratings

#### Memory

Participants rated the CS + (*M* = 1.28, *SD* = 0.99) as more likely to be associated with an US compared with the CS − (*M* =  − 1.01, *SD* = 0.84), *F*(1,64) = 204.38, *p* <.001, *R*^*2*^ =.62, 95% CI [.52,.70], indicating successful differentiation between CS + and CS − during the retrieval task. On average, CS + faces (47.1 ± 19.2%) were more frequently classified as remembered than CS − faces (41.0 ± 14.9%), *F*(1, 64) = 8.10, *p* =.006, *R*^*2*^ =.11, 95% CI [.01,.28]. Neither memory ratings nor the number of remembered faces showed main effects of or interactions with individual ASI levels (all *p*s >.193).

#### Arousal and valence

**Fig. 3:**
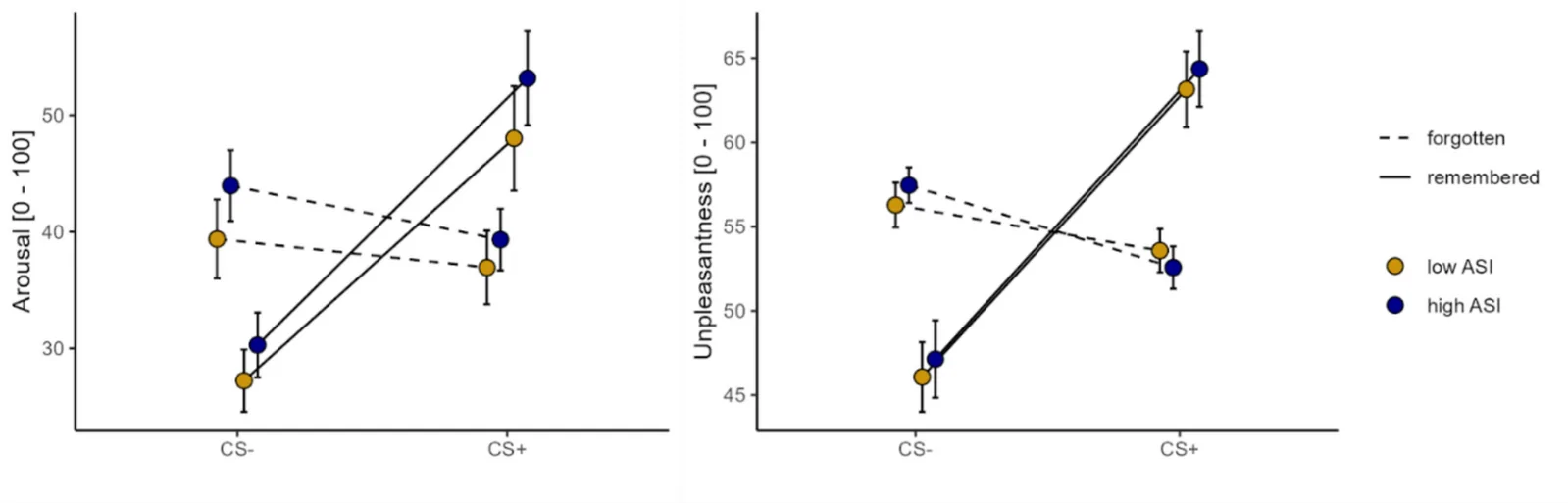
Mean (± SE) arousal and unpleasantness ratings as a function of memory and cue. *Note.* A median split of ASI scores is used solely for illustrative purposes of the continuous ASI covariate

For arousal and valence ratings, the linear mixed models revealed significant interactions of cue and memory, arousal: *F*(1, 192) = 107.22, *p* <.001, *R*^*2*^ =.36, 95% CI [.26,.46], valence: *F*(1, 192) = 76.00, *p* <.001, *R*^*2*^ =.28, 95% CI [.19,.39], suggesting higher arousal and valence ratings for CS + compared with CS −, especially for remembered faces (Fig. 3). No significant effect was found involving ASI levels (all *p*s >.346).

### Pupillary responses

**Fig. 4:**
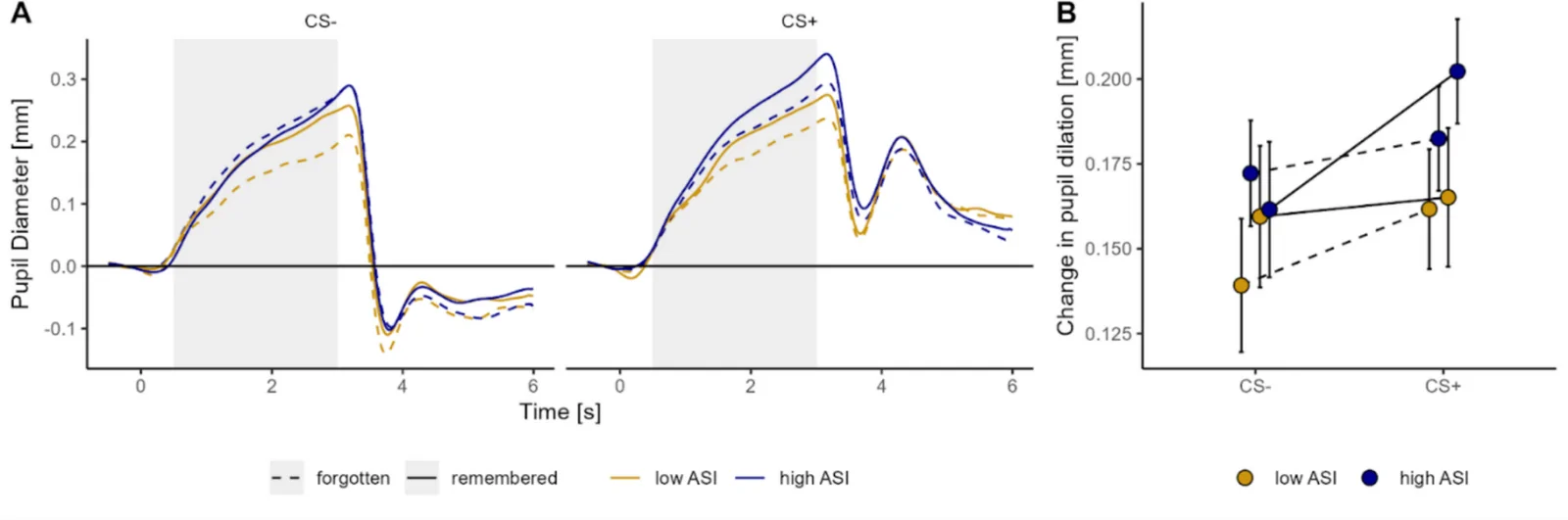
Pupillary responses as a function of cue, memory, and ASI levels. *Note.* **A**) The shaded areas indicate the response interval from 500 to 3,000 ms relative to cue onset. **B**) Mean (± *SE*) pupillary responses across the shaded areas from A). The median split of ASI scores is used solely for illustrative purposes of the continuous ASI covariate

The linear mixed model for pupil responses during the acquisition phase demonstrated a main effect of cue, *F*(1,192) = 18.22, *p* <.001, R^*2*^ =.09, 95% CI [.03,.17], indicating stronger pupil dilation for CS + compared with CS − trials (Fig. 4A). In addition, there was a significant three-way interaction between cue, memory, and individual ASI levels, *F*(1,192) = 7.21, *p* =.008, R^*2*^ =.04, 95% CI [.003,.10] (Fig. 4B). The main effect of memory just failed to reach significance, *F*(1,192) = 3.01, *p* =.084, R^*2*^ =.02, 95% CI [.00,.07]. All other effects were not significant (all *p*s >.151). Follow-up linear mixed models for each cue type revealed a significant effect of memory for CS + trials, *F*(1,64) = 4.45, *p* =.039, R^*2*^ =.07, 95% CI [.001,.22], indicating larger pupil diameter during presentation of CS + associations that were later remembered, but no effect of ASI levels (all *p*s >.099). In contrast, for CS- trials, there was a significant interaction between memory and ASI levels, *F*(1,64) = 5.29, *p* =.025, *R*^*2*^ =.07, 95% CI [.002,.23], indicating decreased differentiation between remembered and forgotten CS- associations as ASI levels increased.

### Event-related potentials to cue onset

#### P300

**Fig. 5:**
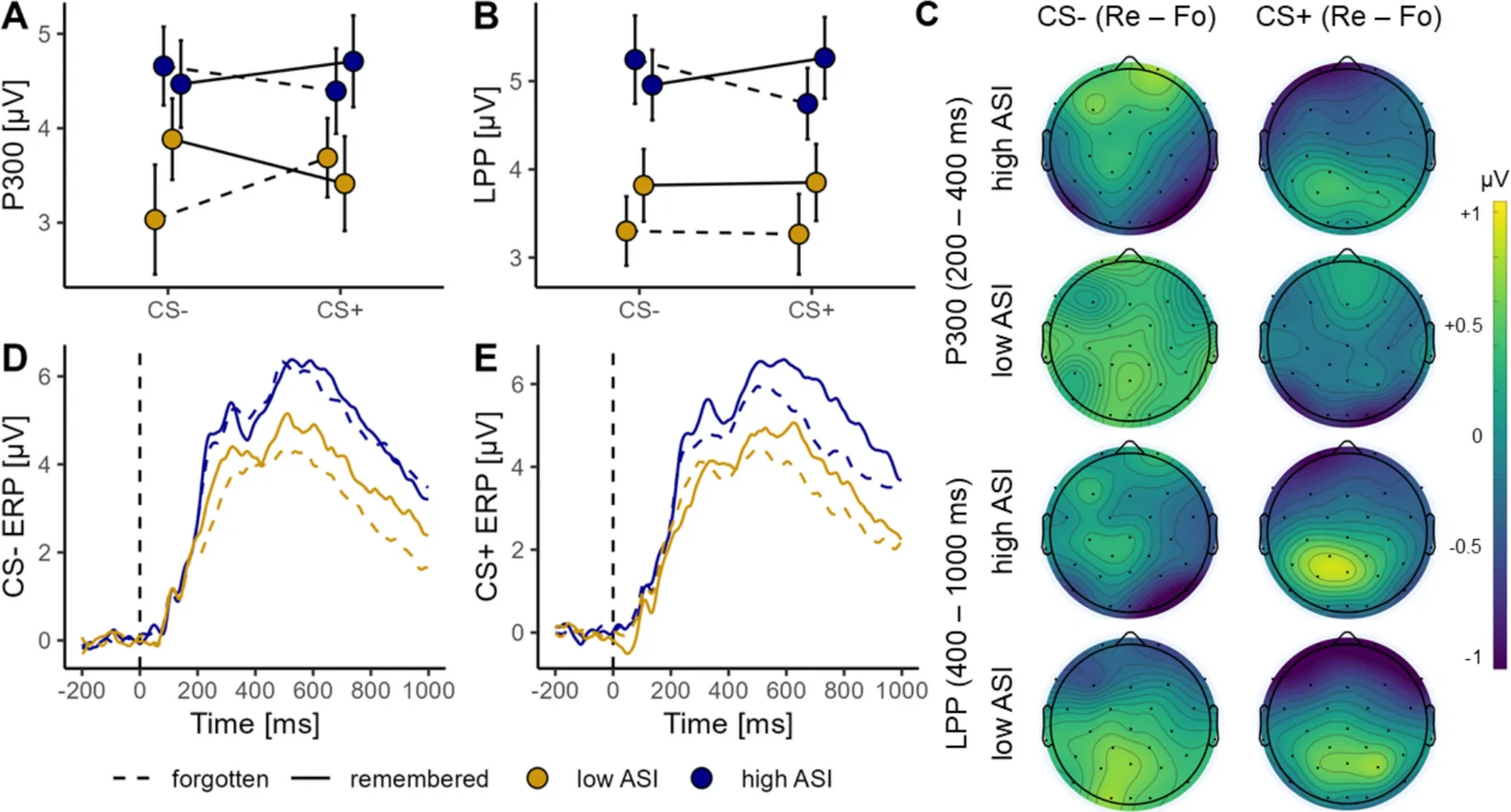
ERPs in response to the cue onsets at Pz. *Note*. Mean (± *SE*) P300 (**A**) and LPP (**B**) responses to the cue onsets at Pz. (**C**) Topographies of the P300 and LPP as a function of CS condition and ASI scores. ERPs in response to the CS − (**D**) and CS + (**E**) at Pz. For ERP plots, data were low pass filtered (20 Hz). The median split of ASI scores is used solely for illustrative purposes of the continuous ASI covariate

The linear mixed model for P300 amplitudes to the cue onsets demonstrated a significant main effect of ASI levels, *F*(1,64) = 5.58, *p* =.021, *R*^*2*^ =.08, 95% CI [.003,.24], suggesting higher P300 amplitudes with increasing ASI levels. Other effects were not significant (*p*s >.174), even though the three-way interaction between cue, memory, and ASI levels was marginally significant, *F*(1,192) = 3.37, *p* =.068, *R*^*2*^ =.02, 95% CI [.00,.07]. This interaction effect might be due to a stronger memory effect for CS- at low compared to high levels of ASI (Fig. 5A). However, the exploratory follow-up linear mixed models, analyzed separately for CS + and CS-, did not reveal any significant interaction effects (all *p*s >.204).

#### LPP

LPP amplitudes to the cue onsets were stronger for remembered compared to forgotten faces, *F*(1,192) = 4.72, *p* =.031, *R*^*2*^ =.02, 95% CI [.001,.08]. In addition, there was a main effect of ASI levels, *F*(1,64) = 8.61, *p* =.005, *R*^*2*^ =.12, 95% CI [.02,.29], indicating generally enhanced LPP amplitudes for higher individual ASI scores (Fig. 5B). Furthermore, there was a marginally significant three-way interaction between cue, memory, and individual ASI levels, *F*(1,192) = 3.25, *p* =.073, *R*^*2*^ =.02, 95% CI [.00,.07]. No other effect reached significance (all *p*s >.137). To exploratively follow up on the marginally significant three-way interaction, we calculated linear mixed models for CS + and CS-, separately. While there was no significant interaction between memory and ASI levels for CS + trials, *F*(1,64) =.59, *p* =.442, the interaction was marginally significant for CS − trials, *F*(1,64) = 3.80, *p* =.055, indicating a slightly stronger memory effect for CS − at low compared with high levels of ASI.

### Event-related potentials to US omission (CS − Offset)

#### P300

**Fig. 6:**
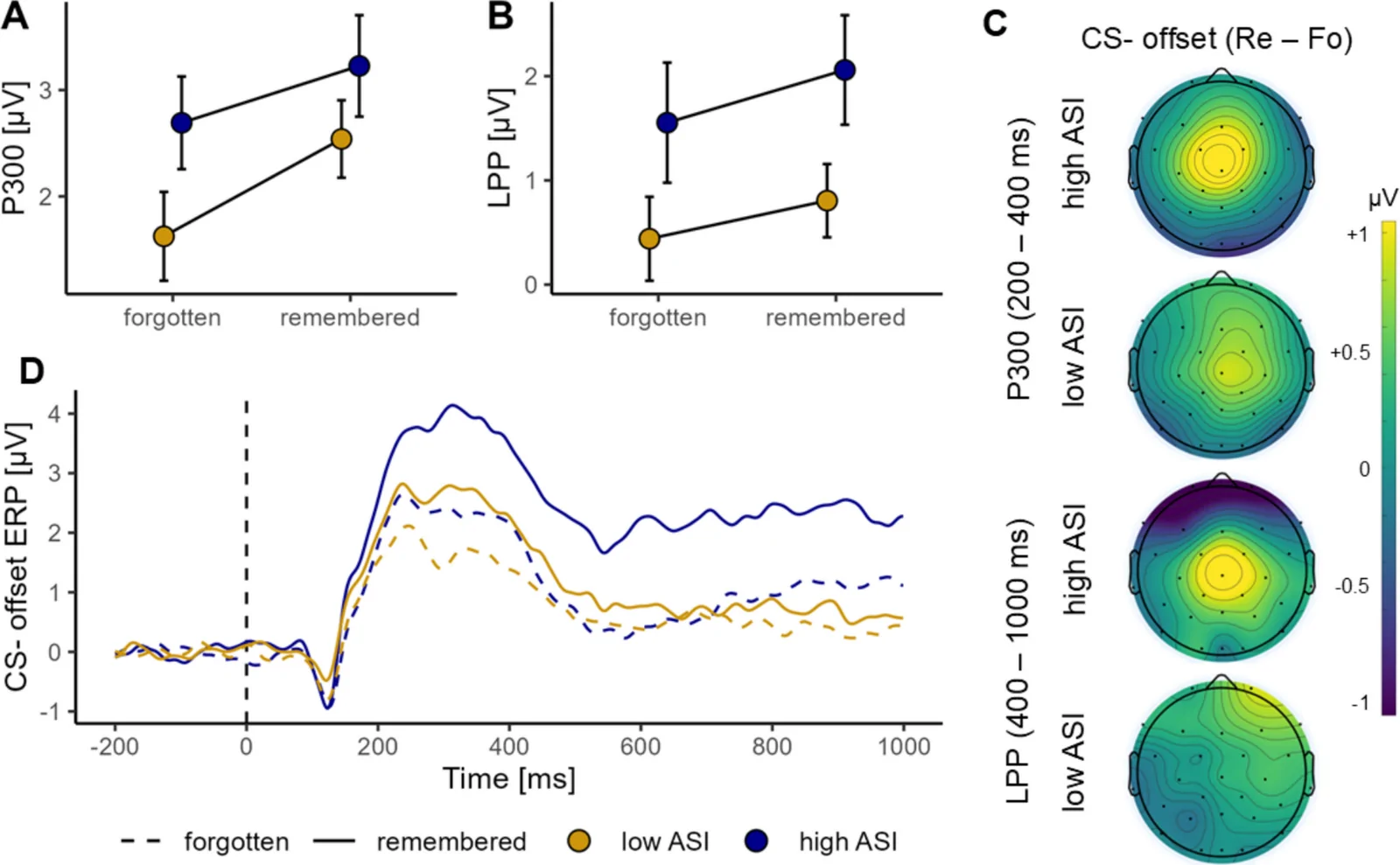
ERPs in response to the CS − offsets at Cz. *Note.* Mean (± *SE*) P300 (**A**) and LPP (**B**) responses to the CS- offsets at Cz. (**C**) Topographies of the P300 and LPP as a function of ASI scores. (**D**) ERPs in response to the CS- offsets at Pz. For ERP plots, data were low pass filtered (20 Hz). The median split of ASI scores is used solely for illustrative purposes of the continuous ASI covariate

The linear mixed model for P300 amplitudes to the cue offset for the CS − (US omission) showed a significant main effect of memory, *F*(1,64) = 8.68, *p* =.004, *R*^*2*^ =.12, 95% CI [.02,.29], suggesting higher P300 amplitudes for remembered compared to forgotten CS − faces (Fig. 6A). In addition, the main effect of ASI levels was marginally significant, *F*(1,64) = 3.47, *p* =.067, *R*^*2*^ =.05, 95% CI [.00,.20]. However, their interaction was not significant, *F*(1,64) = 0.06, *p* =.800, *R*^*2*^ <.01, 95% CI [.00,.08].

#### LPP

Results for LPP amplitudes to the cue offset showed a similar pattern, with marginally significant main effects of memory, *F*(1,64) = 3.79, *p* =.056, *R*^*2*^ =.06, 95% CI [.001,.20], and ASI levels, *F*(1,64) = 3.26, *p* =.076, *R*^*2*^ =.05, 95% CI [.00,.19]. The interaction was not significant, *F*(1,64) = 0.01, *p* =.931, *R*^*2*^ <.01, 95% CI [.00,.08]. LPP amplitudes were slightly higher at the offset of remembered versus forgotten CS − faces, and slightly higher for higher ASI levels (Fig. 6B).

## Discussion

The present study investigated the physiological and electrocortical correlates of memory formation during declarative threat and safety learning, as well as their relationship with individual anxiety sensitivity. To this end, we investigated memory effects in a previously established aversive learning paradigm (Leimeister et al., 2023; Wiemer et al., 2021, 2024), while recording electroencephalogram and pupillometry. Our findings revealed that higher individual ASI levels were not associated with better declarative memory performance, despite being linked to slightly stronger P300 and LPP amplitudes at the onset of both CS + and CS − faces. Additionally, we analyzed electrocortical responses at the offset of CS-, representing a response to the omission of the US. We observed memory-related effects, with enhanced P300 amplitudes, and marginally elevated LPP amplitudes, for faces that were later remembered compared to those that were forgotten. However, these effects were not associated with individual ASI levels. Pupillometry revealed a more nuanced pattern: pupil responses to later forgotten safety cues (CS −) were stronger as individual anxiety levels increased.

These findings suggest that while individuals with higher anxiety sensitivity allocated more attentional and cognitive resources to potentially threatening stimuli (Hunt et al., 2006; Nelson et al., 2015), this heightened processing does not necessarily facilitate declarative memory formation. Consistent with previous research, we observed enhanced LPP amplitudes for subsequently remembered associations between CS and US, indicating that sustained attention to emotional stimuli supports successful memory encoding (Dolcos & Cabeza, 2002; Kiefer et al., 2008). The LPP is a sustained positive potential over parieto-occipital regions that is enhanced for emotionally arousing compared to neutral stimuli (Hajcak et al., 2010, 2013). Similarly, increased LPP amplitudes have been associated with remembered versus forgotten emotional pictures (Dolcos & Cabeza, 2002; Kiefer et al., 2008). However, its precise mechanistic role in memory processes remains unclear. The LPP has been considered an electrocortical marker of sustained attention and perception suggesting that successful memory encoding may result from increased attention to cues, as reflected by enhanced LPPs (Hajcak et al., 2010; Schupp et al., 2000). The LPP is also modulated by top-down processes, likely originating from prefrontal regions (Schupp et al., 2006). In addition, a reciprocal bidirectional link from posterior to frontal regions has been observed for late-latency responses (~ 400 ms) to highly arousing emotional stimuli (Moratti et al., 2011). Consistent with this, our recent fMRI study found that the ventromedial and dorsolateral PFC play a crucial role in the formation of safety memories, potentially by stabilizing the integration of cross-sensory stimulation in visual and somatosensory regions (Wiemer et al., 2024). Taken together, these findings suggest that the LPP may be causally involved in memory formation, possibly by signaling emotional value and motivational significance to frontal regions that support memory processes.

However, the additional overall increase in LPP amplitudes with higher anxiety sensitivity did not translate into enhanced memory performance. One possible explanation is that successful performance in the memory task required encoding not only the face itself but also whether it was paired with an unconditioned stimulus (US). Notably, the memory task did not simply involve recognizing whether a face had been presented during the main experiment, but rather recalling, whether it had been paired with a US or not. This explicit instruction to focus on cue offset may have reduced the importance of cue onset during memory encoding. This interpretation is further supported by our findings on P300 amplitudes at cue onset, which were not associated with successful memory encoding in our task. This suggests that early attentional processing at stimulus onset may have been less relevant for encoding the critical face-US association required for successful memory performance. Furthermore, we found preliminary evidence for aberrant safety learning processes in high levels of anxiety sensitivity. The difference between remembered and forgotten safe associations was marginally weaker in individuals with higher anxiety levels. This indicates that the attentional processing of the faces as indexed by the LPP and P300 amplitudes had a lesser impact on memory formation in high anxiety. Considering that there was no difference in memory performance between anxiety levels, this suggests that safety learning might be compensated by a different mechanism in highly anxious individuals. One possible alternative is that these individuals rely more on automatic, context-based memory processes supported by the hippocampus, rather than salience-driven encoding linked to attention and affect (Fanselow, 2000; Maren et al., 2013). However, this alternative mechanism might be less stable, as previous studies suggest deficient safety learning in anxiety disorders (Kausche et al., 2025). Future studies might as well assess different levels of associative memory, such as after a longer period of time.

In addition, analyses of the electrocortical responses to the CS offsets also revealed evidence for memory-related effects, with both P300 and LPP amplitudes enhanced for faces that were later remembered vs. forgotten, even though the effect for LPP amplitudes was just marginally significant. Consequently, this might suggest that allocating attentional resources to the absence of threat plays a crucial role in safety learning and the formation of safety memories. Notably, the P300 has been associated with sensory prediction error signals (Palidis et al., 2019), and the magnitude of such signals has been linked to subsequent memory formation in humans (Jang et al., 2019). Thus, the presence or omission of the unconditioned stimulus (US) may elicit a prediction error response, reflected in the P300, which ultimately enhances memory formation (Wiemer et al., 2021). However, unlike the onset-related responses, these offset-related ERPs were only marginally modulated by ASI levels. Given the lack of behavioral differences in memory performance as a function of anxiety levels, this finding supports the idea that face-US associations are more critical for successful memory performance than the encoding of the face alone.

It has been suggested that individuals with high anxiety are more sensitive to potential threats, which could lead to heightened emotional responses (Nelson et al., 2015; Stegmann et al., 2019a). This aligns with our findings for the cue on- and offsets, where P300 and LPP amplitudes increased as a function of ASI levels, especially since each face was only shown three times. Indeed, enhanced sensitivity to potential threats may be linked to the allocation of more cognitive resources to processing emotionally relevant stimuli and to amplified responses to stimuli that are perceived as uncertain or unpredictable, as reflected in the LPP and P300, respectively. These findings also support the idea of a general hypervigilance bias in anxious individuals (Bar-Haim et al., 2007; Cisler & Koster, 2010; Mathews et al., 1990; Mogg & Bradley, 1998; Wieser & Keil, 2020).

The findings from the ERPs are further supported by the pupillometry response pattern. Notably, pupil responses were generally stronger for CS + trials compared with CS − trials, indicating successful fear conditioning. This is particularly important as each of the 80 face-US pairs was only presented three times, which reduces the number of learning trials compared to typical fear conditioning studies (Lonsdorf et al., 2017). Moreover, pupil responses were also modulated by memory effects and ASI levels. For CS + trials, the expected main effect of memory was observed, with marginally higher pupil responses for remembered versus forgotten associations. This aligns with the idea that increased arousal is a crucial precursor to memory formation (Bradley et al., 1992; Dolcos & Cabeza, 2002; LaBar & Phelps, 1998; Mather & Sutherland, 2011). However, for CS − trials, this pattern was overlaid with enhanced pupil responses to forgotten CS- associations. It is important to note, that in the present paradigm, memory performance measurement did not target stimulus identity per se, but rather memory for CS–outcome associations. Consequently, a CS − classified as “forgotten” likely reflects a failure to encode the CS − –US omission contingency and may additionally indicate a tendency to misattribute threat to that cue. From this perspective, enhanced pupil dilation during encoding of CS − trials that were later classified as forgotten may reflect heightened autonomic arousal or threat expectancy, which could bias subsequent judgments toward assuming an aversive association. This could suggest that heightened autonomic arousal during safety learning may interfere with memory consolidation, potentially due to increased cognitive load or difficulty disengaging from threat-related processing in participants with higher levels of anxiety (Pessoa, 2009; Robinson et al., 2013).

It is important to note that the elevated pupil responses to forgotten CS − cues in individuals with high anxiety are the only evidence of aberrant safety learning. Given previous findings of dysfunctional safety learning in individuals with anxiety (Kausche et al., 2025), we expected greater differences in CS- responses as anxiety levels increased. A possible explanation for this could be that we primarily investigated sub-clinically anxious participants. Although we explicitly invited individuals who met the criteria for an anxiety-related disorder according to the DSM-5, the final sample included only 16 participants, who met these criteria, which was too little to conduct meaningful subgroup analyses. Another important limitation is that intentional learning may be less modulated by ASI levels compared to incidental learning. We assume that in incidental learning, the influence of top-down modulations is reduced, and the impact of bottom-up modulations from threat-related stimuli is more pronounced. However, we initially chose to investigate intentional learning because the treatment of anxiety disorders typically focuses on intentional, rather than incidental, learning. Finally, for the primary analyses, we relied on dichotomizing the original 8-point confidence scale into “remembered” versus “forgotten” responses, thereby potentially introducing classification noise and obscuring subtle effects. To address this limitation, we conducted complementary exploratory analyses using the continuous US-memory ratings (see supplement). While we were largely able to replicate our main findings; specifically, stronger LPP and P300 amplitudes with higher individual anxiety sensitivity and stronger ERPs for later confidently remembered CS–US associations, the pattern for pupil responses was less robust. Crucially, due to the nature of the paradigm, some cells contained only a small number of analyzable trials. Thus, these results should be interpreted with caution.

In conclusion, the present study explored the electrocortical and pupillometric correlates of memory formation during threat and safety learning, with a focus on the relationship between individual anxiety sensitivity (ASI) and memory performance. Our findings revealed that heightened anxiety sensitivity was associated with larger P300 and LPP amplitudes at the onset of both threat and safety cues suggesting increased attentional allocation indiscriminately to both threat and safety signals. However, despite these enhanced neural responses, higher ASI levels did not correspond to improved memory performance, particularly when it came to the encoding of the association between face and US omission. Accordingly, we observed no effects of anxiety sensitivity on behavioral memory performance. These findings suggest that while participants with high levels of anxiety display a general hypervigilance to threat, this did not interfere with encoding of threat and safety memory during intentional learning. Even though the current study sheds light on the electrocortical correlates of memory processes in general, future work needs to explore how intentional and incidental learning processes differ in individuals with varying levels of anxiety sensitivity. Understanding these processes is crucial for improving treatment strategies for anxiety disorders, which often rely on intentional learning mechanisms to modify maladaptive threat responses.

## Supplementary Information

Below is the link to the electronic supplementary material.

Supplementary file1 (DOCX 259 KB)

## Data Availability

All data and code behind the analyses have been made publicly available at the Open Science Framework and can be accessed at https://osf.io/83zbx/.

## Code availability

All data and code behind the analyses have been made publicly available at the Open Science Framework and can be accessed at https://osf.io/83zbx/.
