# Supplementary material for: Attentional processing in individual anxiety: Electrocortical correlates of threat- and safety-related declarative memory formation
Source: Cogn Affect Behav Neurosci. 2026 May 12;26(5):2297–310. doi: 10.3758/s13415-026-01450-0 (PMC13615150; doi:10.3758/s13415-026-01450-0)
Supplement: Supplementary file 1 [file 13415_2026_1450_MOESM1_ESM.docx]

**Attentional Processing in Individual Anxiety: Electrocortical Correlates of Threat- and Safety-Related Declarative Memory Formation**

Yannik Stegmann^1^, Valentina Glück^1^, Marta Andreatta^2,3^, Julian Wiemer^1^

^1^ Department of Psychology, University of Würzburg, 97070 Würzburg, Germany

^2^ Department of General Psychiatry and Psychotherapy, University Hospital Tübingen, Tübingen, Germany

^3^ Tübingen Center for Mental Health, University Hospital Tübingen, Tübingen, German

**Author Note**

Yannik Stegmann <https://orcid.org/0000-0002-0933-8492>

Valentina Glück <https://orcid.org/0000-0002-7316-1652>

Marta Andreatta <https://orcid.org/0000-0002-1217-8266>

Julian Wiemer <https://orcid.org/0000-0002-7460-0952>

Correspondence concerning this article should be addressed to Yannik Stegmann, Department of Psychology, University of Würzburg, Marcusstraße 9-11, 97070 Würzburg, Germany. Email: [Yannik.stegmann@uni-wuerzburg.de](mailto:Yannik.stegmann@uni-wuerzburg.de), Phone: +49 931 31-84357

**Analyses of excluded participants**

Supplemental table 1. Comparisons between excluded and included participants, revealing no significant differences regarding age, gender distributions, ASI levels, and shock intensities.

|  | Included (n = 66) | | Excluded (n = 18) | | t(82) | | |
| --- | --- | --- | --- | --- | --- | --- | --- |
|  | *M* | *SD* | *M* | *SD* | *t* | *p* | *d* |
| Age | 25.44 | 6.53 | 26.83 | 8.95 | .74 | .463 | .16 |
| Gender | 67 % female | | 77 % female | | .86 | .382 | .19 |
| ASI | 24.30 | 16.73 | 19.06 | 14.75 | 1.21 | .231 | .26 |
| US intensity | 1.32 | 1.31 | 1.48 | 2.26 | .40 | .692 | .09 |

**Analysis of all response measures with US-memory ratings as continuous variable**

To explore the memory findings more thoroughly, we reanalyzed all models using memory scores as a continuous variable rather than a categorical measure. US-memory ratings were collected on an eight-point scale ranging from −4 (“very certain there was no US”) to +4 (“very certain there was a US”), excluding zero. Response measures were analyzed using linear mixed-effects models, including the within-subject factors CS-type (two levels: CS+ vs. CS−) and US-Memory (continuous), and mean-centered ASI scores as a between-subjects covariate. All three main effects and their interactions were specified as fixed effects. Participants were included as random intercepts. LPP and P300 amplitudes in response to US omission were analyzed using the same approach, except that the factor CS-type was not included. Importantly, the number of analyzable trials differed substantially between conditions (see Figure S1A), ranging from an average of approximately 4 to 36 trials per condition. Moreover, several cells were empty. For instance, 21 participants never rated a CS− as +4 (“very certain to be paired with the US”). These imbalances warrant caution in interpreting the results, as underrepresented conditions may introduce bias into the linear mixed-effects models.

*Pupil dilation:* Changes in pupil dilation showed a marginally significant main effect of US-memory, *F*(1,882.65) = 3.58, *p* = .059, *R²* < .01, 95% CI [.00, .02], with stronger pupil dilations for cues later rated as more certainly paired with the US (see Figure S1B). The main effect of CS-type, *F*(1,883.06) = 2.27, *p* = .132, *R²* < .01, 95% CI [.00, .01], and all other effects, *ps* > .405, were not significant.

*Arousal & unpleasantness:* For arousal ratings, the linear mixed models revealed a significant main effect of US-memory, *F*(1,882.31) = 106.79, *p* < .001, *R²* = .11, 95% CI [.07, .15], indicating higher subjective arousal for cues that were more confidently remembered as having been paired with the US (see Figure S1C). All other effects were not significant, *ps* > .184. Similarly, analysis of unpleasantness ratings showed only a significant main effect of US-memory, *F*(1,884.57) = 83.36, *p* < .001, *R²* = .12, 95% CI [.06, .12], but no other effects, *ps* > .292.

*P300:* Linear mixed model analysis of the mean P300 amplitudes to the cue onsets revealed a significant main effect of ASI scores, *F*(1,116.71) = 4.86, *p* = .029, *R²* = .04, 95% CI [.001, .13], indicating larger P300 amplitudes at higher ASI levels. In addition, there was a marginal main effect of CS-type, *F*(1,984.00) = 3.30, *p* = .070, *R²* = .003, 95% CI [.000, .014], which was further qualified by a significant interaction between CS-type and US-memory, *F*(1,984.00) = 3.87, *p* = .049, *R²* = .004, 95% CI [.000, .016]. Specifically, larger P300 amplitudes were observed for CS+ trials that were later rated as more confidently paired with the US and for CS− trials that were later rated as more confidently unpaired (see Figure S1E). Other effects were not significant, *ps* > .185.

*LPP:* Results for the LPP component paralleled those of the P300. The LMM showed a significant main effect of ASI scores, *F*(1,152.67) = 4.66, *p* = .032, *R²* = .03, 95% CI [.001, .103], indicating larger LPP amplitudes at higher ASI levels. In addition, there was a main effect of CS-type, *F*(1,984.00) = 7.37, *p* = .007, *R²* = .007, 95% CI [.001, .022], which was again further qualified by a significant interaction between CS-type and US-memory, *F*(1,984.00) = 9.00, *p* = .003, *R²* = .009, 95% CI [.001, .025]. Other effects were not significant, *ps* > .183. Consistent with the P300 findings, larger LPP amplitudes were observed for CS+ trials that were later rated as more confidently paired with the US and for CS− trials that were later rated as more confidently unpaired (see Figure S1F). This pattern suggests that enhanced P300 and LPP amplitudes were associated with more accurate memory encoding.

*P300 to CS-offset:* For P300 amplitudes to the CS-offset (US-omission), the linear mixed model analysis revealed a main effect of US-memory, *F*(1,460.00) = 12.17, *p* < .001, *R²* = .026, 95% CI [.005, .061], indicating stronger P300 amplitudes to CS- offsets that were subsequently more confidently remembered as having been unpaired with the US (see Figure S1G). The main effect of ASI levels, *F*(1,165.36) = 2.65, *p* = .106, *R²* = .016, 95% CI [.000, .074], and their interaction, *F*(1,460.00) = 2.62, *p* = .133, *R²* = .005, 95% CI [.000, .026], were not significant.

*LPP to CS-offset:* Analysis of the LPP amplitudes to the CS-offsets showed no significant effects, *ps* > .185.

Figure S1. Analysis of all response measures with US-memory ratings as continuous variable. A median split of ASI scores is used solely for illustrative purposes of the continuous ASI covariate.
